# Apple pomace and hempseed cake can reduce methane intensity (CH₄/DMI) and alter the rumen microbiome in dairy cows: a shotgun metagenomic approach

**DOI:** 10.1186/s40104-026-01448-1

**Published:** 2026-06-24

**Authors:** Abdolvahab Ebrahimpour Gorji, Benchu Xue, Tianhai Yan, Tomasz Sadkowski, Xianjiang Chen, Omar Cristobal-Carballo, Steven Morrison, Vahid Razban, Laurence Smith, Sokratis Stergiadis, Katerina Theodoridou, Masoud Shirali

**Affiliations:** 1 Agri-Food and Biosciences Institute, Belfast, BT9 5PX UK; 2 Department of Physiological Sciences, Institute of Veterinary Medicine, Warsaw University of Life Sciences, Warsaw, 02-787 Poland; 3 School of Biological Sciences, Queen’s University Belfast, Belfast, BT9 5DL UK; 4 School of Agriculture, Policy and Development, University of Reading, Reading, RG6 6EU UK

**Keywords:** Apple pomace, Dairy cows, Hempseed cake, Methane mitigation, Rumen microbiome

## Abstract

**Background:**

With growing attention to environmental impacts, the dairy sector is increasingly focused on implementing strategies that lower methane emissions and enhance sustainability while maintaining productivity and economic viability. Utilizing agro-industrial by-products as alternative feed ingredients supports circular economy goals, lowers feed costs, and may benefit rumen fermentation and environmental performance in dairy cows.

**Methods:**

Forty-five mid-lactation Holstein cows were assigned to three diets, Control, Apple Pomace (AP), or Hempseed Cake (HC) for 24 d. Feed intake, milk yield, rumen fermentation, methane emissions, and nutrient use were measured. Rumen samples underwent shotgun metagenome sequencing and bioinformatics analysis to assess microbial and functional changes.

**Results:**

Values are reported as mean ± SEM. Shotgun metagenomic sequencing revealed that both supplements significantly increased the relative abundance of Bacteroidota (AP: 56.7% ± 2.8%, *P* = 0.032; HC: 54.5% ± 3.4%, *P* = 0.048) compared to the Control (48.2% ± 3.1%). Concurrently, Bacillota (formerly Firmicutes) abundance decreased, significantly reducing the Bacillota/Bacteroidota ratio (formerly the Firmicutes/Bacteroidetes ratio) from 0.81 ± 0.06 (Control) to 0.58 ± 0.05 for AP (*P* = 0.012) and 0.64 ± 0.05 for HC (*P* = 0.034). Functional analysis showed that AP increased the abundance of *Segatella bryantii* (2.1-fold, *P* < 0.01), associated with a 1.52-fold enrichment in propionate metabolism pathways (*P* = 0.019). Phenotypically, AP significantly reduced the acetate-to-propionate ratio (AP: 2.41 vs. Control: 4.50; *P* = 0.0075) and methane emissions per unit of dry matter intake (CH_4_/DMI) (AP: 20.33 vs. Control: 24.27 g/kg; *P* = 0.016). HC supplementation upregulated fiber-degrading taxa such as *Xylanibacter ruminicola* (1.6-fold) and enriched xylanase families (GH10: 1.58-fold, *P* = 0.035), alongside a significant reduction in methane intensity (CH_4_/DMI). Total methane output, feed intake, and milk yield were not significantly changed by treatments (*P* > 0.05).

**Conclusions:**

In this short-term (24-d) controlled feeding study in mid-lactation Holstein cows, AP and HC were associated with distinct microbial and functional shifts alongside lower methane intensity, with AP linked to propanoate-related signals and HC to fiber-degrading functions; however, ruminal H_2_ concentration and methanogenesis/hydrogen-metabolism markers were not quantified, so the proposed mechanisms should be interpreted as plausible inferences rather than direct physiological evidence.

**Supplementary Information:**

The online version contains supplementary material available at 10.1186/s40104-026-01448-1.

## Introduction

The international dairy sector faces increasing pressure to improve sustainability without compromising productivity and profitability. Enteric methane emissions, representing 40%−45% of greenhouse gases from ruminant livestock and constituting a loss of 2%−12% of gross energy intake, remain a major environmental and efficiency challenge [1, 2]. Nutritional strategies that modulate rumen fermentation, alter microbial hydrogen flows, or shift methanogenic activity have shown potential to reduce these emissions [3]. However, many proposed interventions lack economic feasibility or long-term applicability in commercial systems.

One promising approach is the incorporation of agro-industrial by-products into dairy cow diets. Valorizing such residues aligns with circular economy principles by reducing waste, lowering feed costs, and potentially improving both environmental and production outcomes [4, 5]. Among these materials, apple pomace (AP), a fibrous and polyphenol-rich residue comprising ~25% of the original fruit mass [6], and hempseed cake (HC), a protein-dense by-product of hemp oil extraction (30%−40% crude protein) [7], represent underexplored feed alternatives. Their inclusion could influence rumen fermentation, nutrient use efficiency, and enteric methane generation, yet their nutritional and microbial impacts in lactating dairy cows remain poorly defined.

The rumen microbiome, a complex community of bacteria, archaea, fungi, and protozoa, is central to feed degradation, volatile fatty acid (VFA) production, microbial protein synthesis, and methanogenesis [8, 9]. Dietary changes can rapidly shift its taxonomic structure and functional activity, influencing both animal productivity and environmental outputs. Modern high-throughput sequencing, particularly shotgun metagenomics, now enables comprehensive characterization of rumen microbial communities beyond culture-based or amplicon approaches. This technique captures the full genetic potential of the microbiome and allows functional annotation using KEGG, COG, and CAZy databases, revealing metabolic pathways and enzymes linked to fiber breakdown, protein metabolism, and methane formation [10–12]. Throughout this investigation, we use updated phylum nomenclature (Bacillota for former Firmicutes; Bacteroidota for former Bacteroidetes); accordingly, we refer to the Bacillota/Bacteroidota ratio (formerly the Firmicutes/Bacteroidetes, F/B, ratio where relevant).

Recent metagenomic studies have demonstrated strong associations between rumen microbial functions, feed efficiency, nitrogen use, and milk protein production [13, 14]. Moreover, dietary composition often exerts a greater influence on rumen microbiome structure than host genetics, highlighting the potential of microbiome-based nutritional interventions [15]. Large-scale multi-omics studies further show that methane mitigation strategies (e.g., 3-NOP supplementation) remodel microbial fermentation pathways and hydrogen sinks in species-resolved detail [16]. Such insights support emerging “Rumen Microbiome Nutriomics” approaches aimed at precision dietary modulation of microbial phenotypes [17–20].

Despite growing interest, the effects of AP and HC on the rumen metagenome, and their consequences for nutrient utilization, milk production, and methane intensity, have not been previously characterized. Limited evidence exists for HC in dairy cows [21], almost no data are available for AP in lactating animals. Importantly, no study to date has integrated detailed production measurements, nutrient digestibility, methane emissions, and shotgun metagenomic profiling to evaluate these by-products.

This experiment explored the potential of two agro-industrial by-products, AP and HC, as sustainable feed components for mid-lactation dairy cows. By combining shotgun metagenomic sequencing with measurements of dry matter intake, milk yield, rumen fermentation, nutrient digestibility, and methane emissions, this study aims to elucidate how these by-products modulate rumen microbial ecology and functional pathways, and to assess their potential to improve both environmental and production outcomes.

## Material and methods

### Experimental design

This experiment was designed to assess the effects of feeding AP and HC, byproducts from the UK food processing industry, on feed intake, milk production, rumen fermentation, nutrient use efficiency, and methane emissions in mid-lactation Holstein dairy cows. The experimental design was published previously [22]. Briefly, in that study, 45 multiparous Holstein dairy cows (*n* = 15 per group) were used. Cows received three dietary treatments during the experiment (Control, AP, and HC) and were introduced after 90 days of calving, balanced by parity, body weight, and average milk yield, and housed at the Agri-Food and Biosciences Institute (AFBI), Hillsborough. The study lasted 24 d and consisted of a 17-day adaptation and measurement period followed by a 7-day collection phase. The diets included: (1) Control, grass silage with standard concentrate; (2) AP, 14.3% apple pomace/straw silage; and (3) HC, 10% hempseed cake. Primary forages consisted of 45.7% grass silage and 4.3% barley straw, with Thompson concentrate pellets (30%−40%) and milking parlour supplements included. Pellets were provided via GreenFeed units (1 kg DM/cow/d) and the milking parlour (1 kg DM/cow/d). GreenFeed units were calibrated following the manufacturer’s recommendations (http://greenfeed.c-lockinc.com). GreenFeed is equipped with a head position sensor and gas emission data are rejected when the cow’s head position criteria are not met. Each cow was allowed a maximum of 4 visits in 24 h, with a 4-h interval between visits. A total of 1,030 GreenFeed visits (an average of 3.3 visits/cow/d) were collected and processed from the study and a valid GreenFeed visit was defined as a record in which the cow was correctly identified and remained in the sampling hood for at least 2 min [23]. During the metabolism phase, cows received an additional 2 kg DM of pellets daily as a milking top-up [22].

### Sampling and measurements

Rumen fluid samples were collected from 45 multiparous Holstein dairy cows (*n* = 15 per group) on d 24 of the period, 2 h after feeding, for measurement of pH, VFA, ammonia-N, and microbial/omics analyses. Rumen sampling was conducted once at the end of the experimental period (d 24), following the 17-d adaptation, to provide an end-point snapshot of the diet-associated rumen microbiome under standardized post-feeding conditions while minimizing repeated invasive sampling and disturbance during the methane and metabolism measurements [22]. Approximately 30 mL of blood was collected from the coccygeal vein 3 h post-feeding on d 24 to assess biochemical markers, including total protein, albumin, globulin, urea, β-hydroxybutyrate, and enzymes (GLDH, GGT, GPX), as well as minerals. Dry matter intake (DMI) was recorded for each cow during the study and summarized over the 7 d collection phase (d 18–24) by calculating feed offered minus refusals on a dry matter basis. Milk yield (MY) was recorded at each milking using the herd recording/parlour system and summarized over the same collection period. Methane emissions were measured using the GreenFeed system during the study period, and methane intensity was expressed as CH₄ per unit of DMI (g/kg DMI). All phenotypic variables shown in this manuscript correspond specifically to Period 1 of the previously published three‑period experiment.

### DNA extraction

Genomic DNA was extracted from approximately 200 mg of homogenized rumen samples (combined fluid and solid content) from 45 multiparous Holstein dairy cows (*n* = 15 per group) using the BayBiopure Magnetic Stool Nucleic Acid Kit (BayBio Tech, Hangzhou, China) following the manufacturer's protocol. The procedure involved bead-beating lysis followed by high-temperature incubation (95 °C, 10 min) to ensure efficient cell disruption. Magnetic bead-based purification with multiple wash steps was employed to remove PCR inhibitors, including humic substances and polyphenols prevalent in rumen samples. Purified DNA was eluted in 50 μL low-salt buffer and quantified using a NanoDrop spectrophotometer and accepted for sequencing only when the A_260_/A_280_ ratio was 1.8–2.0 and integrity was confirmed by 1.0% agarose gel electrophoresis. Only samples meeting these quality standards were retained and stored at −80 °C until metagenomic sequencing.

### Bioinformatics analysis

Rumen samples underwent shotgun sequencing on an Illumina NovaSeq X Plus platform (2 × 150 bp), generating approximately ~60 million paired-end reads per sample. Sequencing depth and annotation yield are summarized as: (i) the number of read pairs retained per sample after adapter/quality trimming, (ii) the fraction of reads removed as bovine DNA by Bowtie2 mapping to the *Bos taurus* genome, and (iii) the fraction of non-host reads classified by Kraken2 at each taxonomic rank (domain → species); these summary statistics are reported in the Results (first paragraph of ‘Taxonomic diversity and differential abundance’). Raw sequencing data underwent a comprehensive pre-processing workflow. Raw sequencing data were assessed with FastQC and MultiQC, trimmed for adapters and low-quality bases using Trimmomatic, and then filtered for host (bovine) DNA contamination was removed by mapping reads to the *Bos taurus* genome with Bowtie2, and only unmapped reads were retained for downstream microbial profiling. We did not additionally subtract reads by mapping to feed/plant reference genomes because the experimental diets contained multiple plant-derived ingredients (silage, straw, concentrate pellets, and the by-products AP/HC), for which no single representative reference genome exists. Moreover, subtraction against multiple plant genomes can introduce reference-driven bias and may inadvertently remove true microbial reads due to conserved/homologous regions. Therefore, we restricted subtraction to host DNA and retained non-host reads for downstream analysis; the proportion of residual plant-derived DNA within the non-host read set was not explicitly quantified and is considered a study limitation. We did not perform targeted quantification of methanogenesis marker genes (e.g., methyl coenzyme M reductase (*mcrA*)) from metagenomic reads, nor did we conduct qPCR-based *mcrA* quantification; thus, methanogen functional potential/activity was not directly quantified in this study. In addition to read-level taxonomic profiling, non-host paired-end reads were assembled de novo using MEGAHIT with a minimum contig length of 1,000 bp. Reads were mapped back to assembled contigs using BWA-MEM, and sorted BAM files were processed with jgi_summarize_bam_contig_depths to generate contig coverage depth profiles. Metagenome-assembled genomes (MAGs) were reconstructed by binning contigs using MetaBAT2, and MAG quality (completeness/contamination) was assessed using CheckM (lineage workflow). The full pipeline script is provided in the Additional file 1. Taxonomy was classified with Kraken2 [24], using the Kraken2 standard database (build: k2_standard_20250714), while functional annotation involved Prodigal [25] for open reading frame prediction and eggNOG-mapper/DIAMOND [26] for alignment against eggNOG; KEGG [27], COG [28], and CAZy via dbCAN [29] databases. KEGG pathway profiles were summarized from the functional annotation output to generate KEGG pathway abundance tables used for downstream differential analyses and visualization. Resulting abundance tables were combined with phenotypic metadata (methane output, milk yield, DMI, VFA, digestibility) using R (v4.2.0)(packages: tidyverse [30] for data manipulation and visualization, phyloseq [31] for microbiome data integration, diversity, and ordination, vegan [32] for diversity metrics, community ecology, and multivariate statistics, DESeq2 [33] for differential abundance testing between groups). Analyses included diversity assessments, differential abundance testing (FDR < 0.05), and visualizations via heatmaps and plots. Although MAGs were reconstructed and quality-checked (CheckM completeness/contamination), this manuscript focuses on (i) read-level taxonomic profiling (Kraken2) and (ii) gene-centric functional annotation (KEGG/COG/CAZy) because these approaches directly address the primary study aims and allow consistent quantification of microbial functions across all cows. A full genome-resolved (MAG-based) comparative analysis across treatments (including dereplication of bins across animals, species-level MAG taxonomy assignment, MAG abundance estimation across samples, and MAG-centric functional gene comparisons) was not performed here and is therefore not reported, because it would require additional dedicated analyses beyond the scope of the present manuscript. Taxonomic ranks and label conventions.

Taxonomic profiles from Kraken2 were summarized at multiple ranks, depending on the analysis (phylum, order, family, genus, and species, only when confident resolution was available). Species names are reported only when reads were confidently assigned at the species level; otherwise, taxa are summarized at genus or higher rank to avoid over-interpretation. In the text and figures, ‘Genus sp.’ indicates an unspecified or unresolved single species within a genus, whereas ‘Genus spp.’ indicates a pooled group of multiple unresolved species within the same genus.

### Statistical analysis

Unless otherwise stated, results are reported as mean ± standard error of the mean (SEM), and error bars in figures represent SEM. Fermentation and production outcomes (e.g., VFA traits, A/P ratio, CH_4_ intensity, DMI, and milk yield) were analyzed using a linear model with dietary treatment as the fixed effect and cow as the experimental unit. Least-squares means (LS-means; estimated marginal means) and associated SEM were obtained for each treatment, and pairwise comparisons among treatments were performed with Tukey adjustment for multiple comparisons. *P*-values reported for phenotypic traits correspond to treatment-effect tests and/or Tukey-adjusted pairwise contrasts from this model.

#### Alpha diversity analysis

Alpha diversity was assessed using multiple complementary indices to capture different aspects of within-sample diversity, including Shannon (diversity), Simpson (or inverse Simpson) (dominance/evenness), and observed richness (number of observed features; and/or Chao1 as a richness estimator). These indices were calculated in R (v4.2.0) using phyloseq/vegan. Treatment effects on each alpha diversity index were assessed using one-way ANOVA with treatment as a fixed effect, followed by Tukey’s HSD for post-hoc comparisons when appropriate (*P* < 0.05). This manuscript does not include formal multivariable microbiome–phenotype modeling or feature-selection analyses (e.g., Spearman correlation screening, random forest models, or constrained ordination such as RDA/CCA). Therefore, links between diet-responsive taxa/pathways and phenotypes are described descriptively as associations and interpreted cautiously (non-causal), particularly given the single end-point sampling and the compositional nature of relative-abundance metagenomic data.

#### Beta diversity analysis

Beta diversity was assessed using vegan package (adonis for PERMANOVA, Bray–Curtis dissimilarity, and PCoA visualization) calculated from relative abundance data. Principal coordinates analysis (PCoA) was performed to visualize differences in community structure. Statistical significance of treatment effects on community composition was tested using PERMANOVA [34] (adonis function in vegan) with 9,999 permutations.

#### Individual taxa analysis

The differential abundance of bacterial taxa between treatments was assessed using DESeq2 for Differential abundance testing between groups (FDR < 0.05) based on treatment means derived from taxonomic profiles using tidyverse and phyloseq R packages software. For each taxon, pairwise comparisons (AP vs. Control, HC vs. Control) were performed. *P*-values were calculated and significance was determined using standard thresholds: ^***^P < 0.001, ^**^P < 0.01, ^*^P < 0.05, ^†^*P* < 0.1. Fold changes were calculated as the treatment mean divided by the control mean.

#### Effect size reporting

In addition to *P*-values, we report effect sizes to aid biological interpretation. For continuous phenotypes, effect sizes are presented as absolute mean differences (Δ; treatment − Control) and percent changes relative to Control; where applicable, standardized mean differences (Hedges’ g) are provided. For compositional microbiome outcomes (relative abundances), effect sizes are presented primarily as absolute differences (percentage points) and relative changes (%), and standardized mean differences are reported only as supportive descriptors.

## Results

### Rumen microbial diversity and community structure

#### Alpha diversity

Shannon diversity index values were compared across three treatment groups (Control, AP, HC) to assess bacterial community diversity. One-way ANOVA revealed no statistically significant difference in Shannon diversity among the treatment groups (*F*(2,42) = 1.07, *P* = 0.35). Mean Shannon indices were 5.27 ± 0.28 (Control), 5.42 ± 0.31 (AP), and 5.48 ± 0.29 (HC). The treatment mean square (MS = 0.164) was comparable to the residual mean square (MS = 0.153), indicating that within-group variation exceeded between-group differences. In addition to Shannon diversity, other alpha diversity metrics (richness and dominance-sensitive indices) were also evaluated and showed no significant differences among treatments (*P* > 0.05; reported in Additional file 2).

**Fig. 1:**
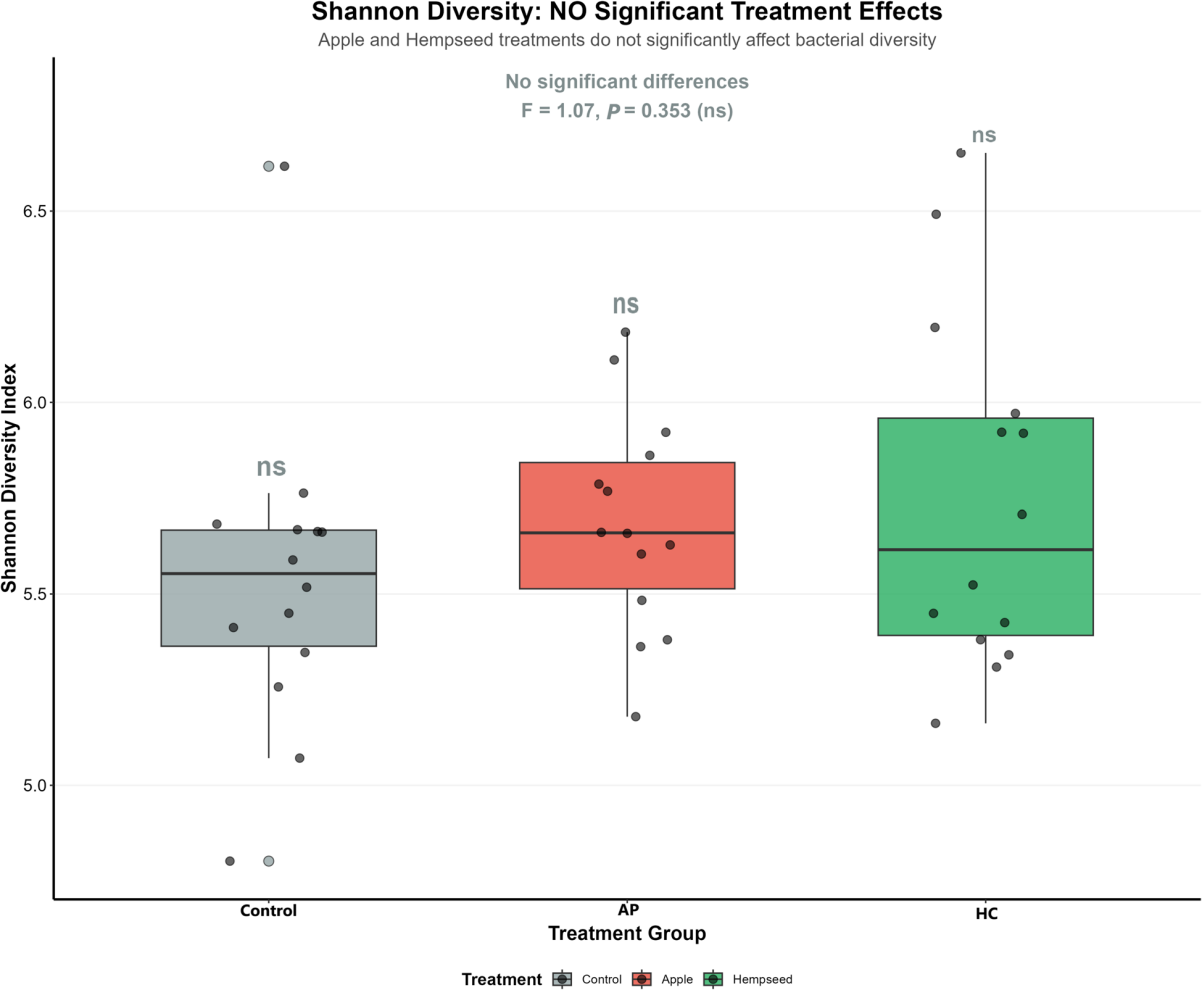
Alpha diversity of rumen bacterial communities across dietary treatments. Shannon diversity indices revealed no significant differences in microbial diversity among the Control, AP, and HC treatments in mid-lactation Holstein dairy cows (45 multiparous Holstein dairy cows; *n* = 15 per group). Box plots display median (center line), interquartile range (box), and range (whiskers). Apple Pomace (AP) red color, Hempseed Cake (HC) green color. Treatment colors are consistent across all figures (Control, AP, HC) to ensure visual comparability

Post-hoc pairwise comparisons using Tukey's HSD test revealed no significant differences between any treatment combinations. AP showed a mean difference of 0.149 compared to Control (95% CI: −0.211 to 0.509, *P*-adj = 0.576), while HC differed from Control by 0.210 (95% CI: −0.150 to 0.571, *P*-adj = 0.339). The comparison between HC and AP yielded a mean difference of 0.061 (95% CI: −0.299 to 0.422, *P*-adj = 0.909). All confidence intervals crossed zero, further supporting the null hypothesis of no treatment effect on alpha diversity (Fig. 1).

#### Beta diversity

PCoA of Bray–Curtis dissimilarity matrices demonstrated partial separation of microbial community structures among the three dietary treatments. PERMANOVA detected statistically significant but modest compositional shifts in ruminal microbiome beta diversity (*F*(2,42) = 5.23, *R*^2^ = 0.185, *P* = 0.001). Although treatments explained 18.5% of the community variance, principal coordinates analysis revealed substantial overlap between treatment groups, indicating subtle rather than dramatic compositional restructuring. The modest *R*^2^ value, combined with visual overlap in ordination space, suggests that individual animal variation (81.5% of total variance) has a predominant influence over dietary treatment effects. Despite limited visual separation, betadisper confirmed homogeneous dispersions (*F*(2,42) = 1.83, *P* = 0.173), validating centroid-based differences. These results indicate statistically detectable but biologically subtle shifts in the microbiome, consistent with functional pathway enrichment patterns observed in KEGG analysis (Tables 1 and 2).

**Table 1:** PERMANOVA analysis of treatment effects on ruminal microbiome beta diversity

Source	Df	SS	MS	*F*	*R*^2^	*P*-value	Significance
Treatment	2	1.234	0.617	5.23	0.185	0.001	***
Residual	42	5.321	0.118	-	0.815	-	-
Total	44	6.555	-	-	1	-	-

PERMANOVA was performed using Bray–Curtis dissimilarity matrices with 9,999 permutations

*df* Degrees of freedom, *SS* Sum of squares, *MS* Mean squares, *F* *F*-statistic, *R*^2^ Coefficient of determination (proportion of variance explained). ^***^P < 0.001

**Table 2:** Pairwise PERMANOVA comparisons between dietary treatments

Comparison	*F*-statistic	*R*^2^	*P*-value	Interpretation
Control vs. AP	7.82	0.213	0.003^**^	Significantly different
Control vs. HC	6.34	0.168	0.012^*^	Significantly different
AP vs. HC	3.45	0.094	0.067^†^	Marginally different

Pairwise comparisons performed using Bray–Curtis dissimilarity with 9,999 permutations. *R*^2^ represents the proportion of variance explained by treatment within each comparison. ^**^P < 0.01; ^*^P < 0.05; ^†^*P* < 0.1 (marginal significance)

#### Taxonomic diversity and differential abundance

Shotgun metagenomic analysis of rumen samples (*n* = 15 per treatment) revealed microbiota affiliated with 25 phyla, 90 orders, 248 families, and 2,170 genera (Additional file 3 and Additional file 4). Comprehensive taxonomic profiling demonstrated treatment-induced shifts at multiple hierarchical levels, with Bacteroidota expansion and Bacillota reduction as dominant responses. Because reference-based classification of short reads is constrained by the availability/representation of rumen genomes in current databases, not all reads can be resolved to species level; therefore, we report results primarily at genus (or higher) rank and use ‘sp.’/‘spp.’ labels as defined in the Methods.

### Phylum-level composition

**Fig. 2:**
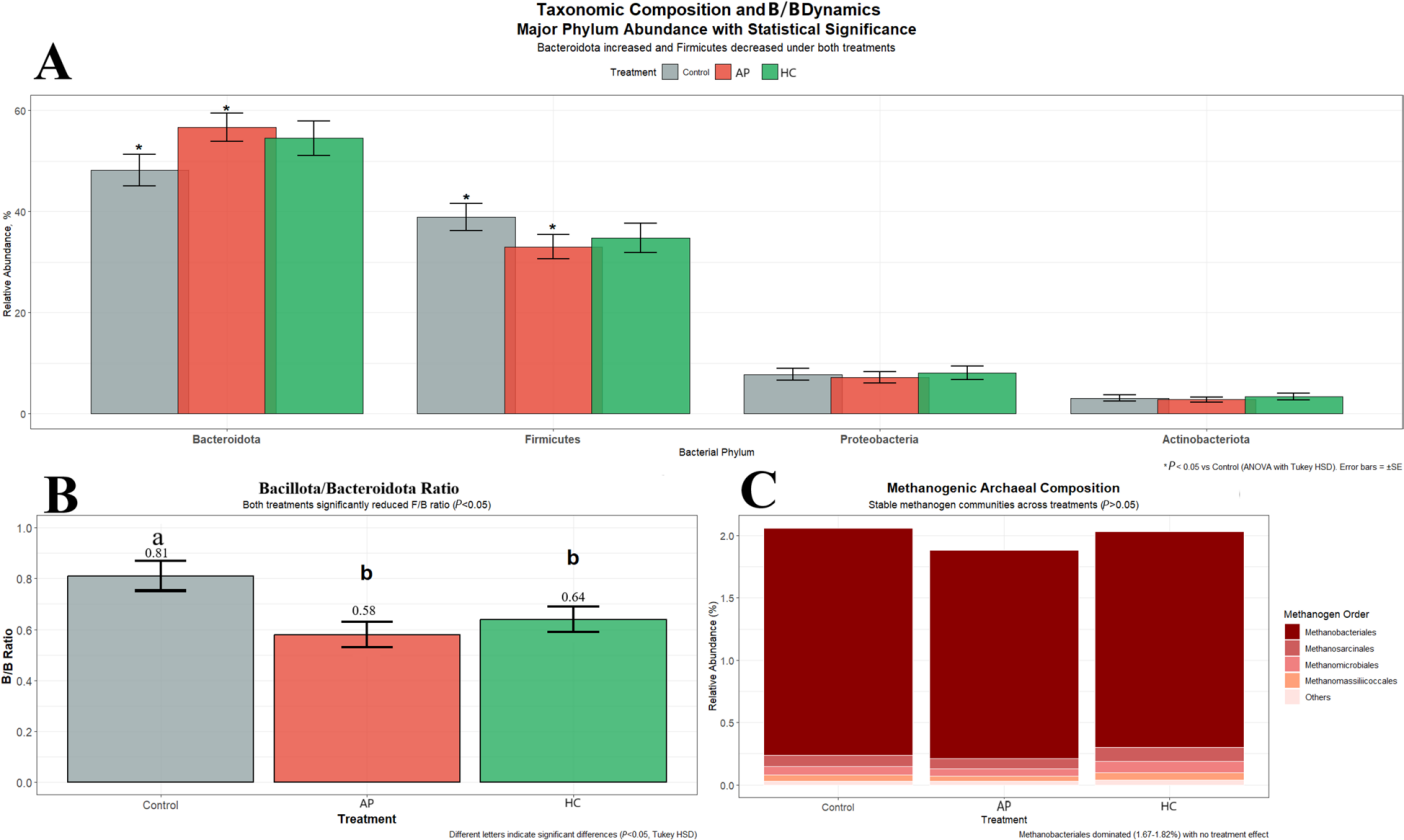
Phylum-level taxonomic composition and community dynamics. **A** Relative abundance of major bacterial phyla showing significant enrichment of Bacteroidota under AP (56.7%, *P* = 0.032) and HC (54.5%, *P* = 0.048) treatments compared to Control (48.2%). Bacillota decreased under both treatments (*P* < 0.05). Error bars represent ± SEM. **B** Bacillota/Bacteroidota ratios demonstrate significant reductions under both agro-industrial by-product treatments (*P* < 0.05). Different letters indicate significant differences (Tukey HSD). **C ***Methanogenic archaeal* composition dominated by Methanobacteriales (1.67%−1.82%), showing no treatment effects (*P* > 0.05), indicating stable methanogen communities. Forty-five multiparous Holstein dairy cows (*n* = 15 per group). Apple Pomace (AP) red color, Hempseed Cake (HC) green color. Taxa in panels (**A**) and (**C**) are shown at the phylum and order level, respectively; the ratio in panel (**B**) is derived from phylum-level relative abundances. Treatment colors are consistent across all figures (Control, AP, HC) to ensure visual comparability

At the phylum level, Bacteroidota (formerly Bacteroidetes), Bacillota, *Proteobacteria*, and Actinobacteriota dominated across all treatments (Fig. 2A). Bacteroidota constituted 48.2% ± 3.1% (Control), 56.7% ± 2.8% (AP), and 54.5% ± 3.4% (HC) of total rumen microbiota. AP significantly increased Bacteroidota by 8.5 percentage points (*P* = 0.032), while HC increased it by 6.3 points (*P* = 0.048). Conversely, Bacillota decreased from 38.9% ± 2.7% (Control) to 33.0% ± 2.4% (AP, *P* = 0.041) and 34.8% ± 2.9% (HC, *P* = 0.039). Effect sizes (absolute percentage-point differences, percent changes, and standardized mean differences where applicable) for key taxa are summarized in Additional file 5.

The Bacillota/Bacteroidota ratio (formerly termed the Firmicutes/Bacteroidetes, F/B, ratio) was significantly reduced under both treatments: 0.81 ± 0.06 (Control), 0.58 ± 0.05 (AP, *P* = 0.012), and 0.64 ± 0.05 (HC, *P* = 0.034; Fig. 2B). This shift toward a lower Bacillota/Bacteroidota ratio is reported here as a descriptive phylum-level compositional change. Given the coarse and compositional nature of phylum ratios, it should not be interpreted as a direct causal driver of methane mitigation; instead, the functional and genus/species-level changes reported below (e.g., propionate-associated taxa/pathways and CAZy shifts) provide a more biologically informative basis for interpretation (Fig. 2).

**Fig. 3:**
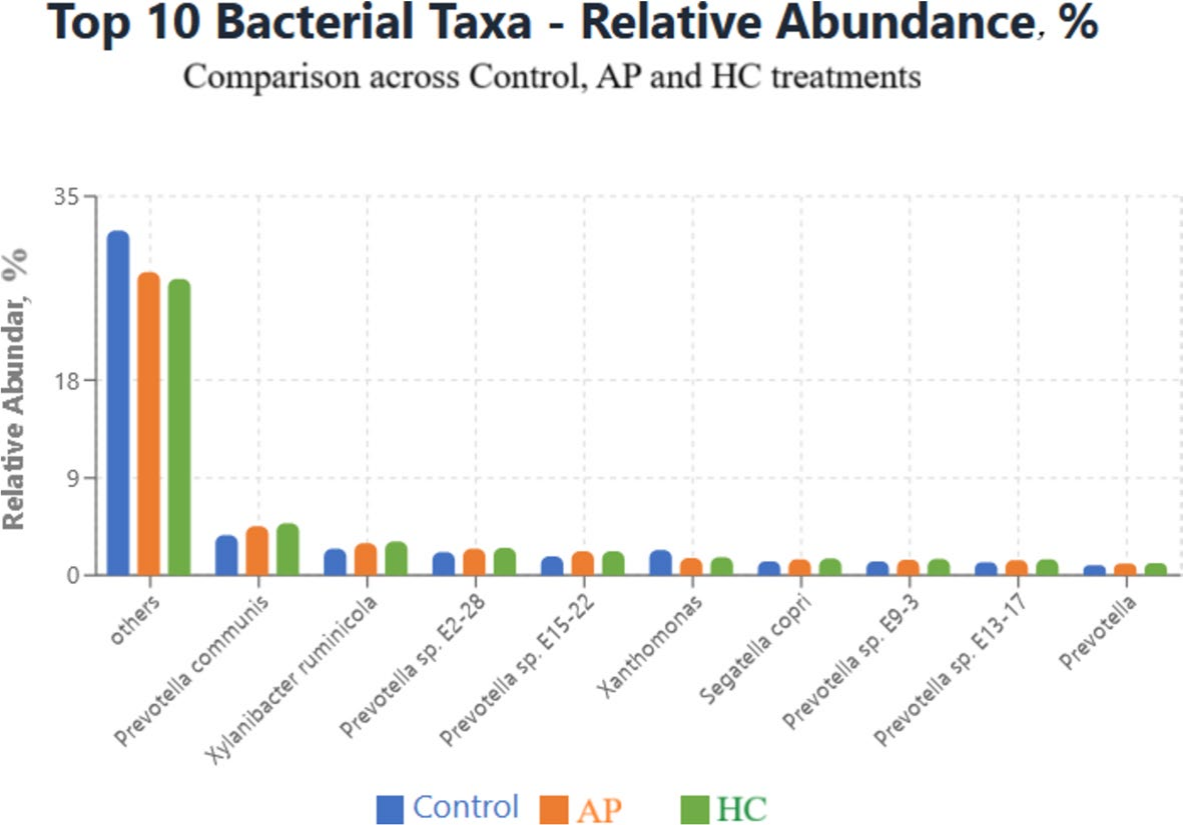
Individual taxonomic composition of rumen bacterial communities. Relative abundance of bacterial phyla across individual cow samples within each dietary treatment group (Control, AP, HC). Each stacked bar represents a single sample, showing the proportional contributions of the dominant phyla to the total microbial community. Apple Pomace (AP) red color, Hempseed Cake (HC) green color. Taxa are shown at the phylum level (relative abundance, %). Treatment colors are consistent across all figures (Control, AP, HC) to ensure visual comparability

A strong negative association between Bacillota and Bacteroidota (formerly Firmicutes/Bacteroidetes nomenclature) was observed in relative abundance data across samples. Because relative abundances are compositional (summing to 100%), this pattern may reflect a mathematical trade-off rather than a direct biological interaction between the two phyla; therefore, it is reported descriptively rather than interpreted as antagonism. Minor phyla included Proteobacteria (7.2%−8.1%), Actinobacteriota (2.8%−3.4%), Euryarchaeota (1.8%−2.1%), Verrucomicrobiota (0.4%−0.6%), Spirochaetota (0.3%−0.5%), and Fibrobacterota (0.2%−0.4%), none showing significant treatment effects (*P* > 0.05 for all) (Fig. 3).

### Order-level dynamics

Bacteroidales and Eubacteriales constituted the two largest orders, representing 42.3%−49.8% and 22.1%−24.6% of microbiota, respectively (Fig. 2C). AP increased Bacteroidales to 49.8% ± 3.2% (*P* = 0.018 vs. Control: 42.3 ± 2.9%), while HC showed intermediate enrichment at 47.5% ± 3.6% (*P* = 0.042). Eubacteriales, Selenomonadales (8.2%−9.7%), Fibrobacterales (2.8%−3.4%), Lachnospirales (2.1%−2.8%), and Oscillospirales (1.8%−2.4%) remained stable (all *P* > 0.05).

Among archaea, Methanobacteriales dominated (1.67%–1.82% of the metagenome), with no significant treatment effects (*P* = 0.23), despite an 8.2% reduction trend under AP. Other methanogenic orders (Methanosarcinales, Methanomicrobiales, Methanomassiliicoccales) accounted for < 0.11% collectively and remained stable across treatments. In this dataset, archaeal signals were low relative to bacterial reads and, consistent with conservative reference-based classification, methanogens were therefore summarized at higher taxonomic ranks (order/family) rather than forcing genus/species-level assignments. Importantly, these DNA-based relative-abundance results do not quantify methanogenesis functional genes (e.g., *mcrA*) or pathway activity/expression; thus, the absence of a detectable difference in methanogen relative abundance should not be interpreted as evidence of unchanged methanogenesis capacity.

### Family-level patterns

Prevotellaceae dominated at 28.3% ± 2.4% (Control), 36.8% ± 2.9% (AP, *P* = 0.006), and 34.2% ± 3.1% (HC, *P* = 0.018), consistent with enhanced propanoate metabolism. Other major families, Lachnospiraceae (12.1%−14.3%), Ruminococcaceae (8.7%−10.2%), Oscillospiraceae (5.4%−6.8%), Methanobacteriaceae (1.70%−1.80%), and Fibrobacteraceae (2.7%−3.3%), showed no treatment effects (all *P* > 0.28), supporting functional redundancy.

### Genus-level differential abundance

**Fig. 4:**
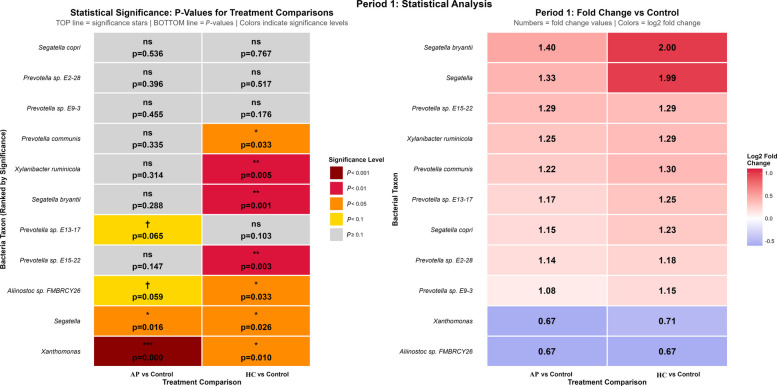
Statistical and functional analysis of gut bacterial taxa. The left heatmap shows the statistical significance (*P*-values) of bacterial abundance differences between treatments (AP vs. Control and HC vs. Control). Each cell is color-coded by significance level (gray = not significant, yellow/orange/red = increasing significance), with stars and *P*-values indicating statistical thresholds (^†^*P* < 0.1; *P < 0.05; ^**^P < 0.01; ^***^P < 0.001). The right heatmap depicts fold changes (vs. Control) for each bacterial taxon, with color intensity representing the log_2_ fold change (red = increased abundance, blue = decreased abundance). Taxa are shown at the genus level, except where explicitly labeled as “sp.” or “spp.”, which indicate unresolved species within a genus (“sp.”) or pooled unresolved species (“spp.”), used when species-level classification is not supported by reference-based read assignment. Treatment colors are consistent across all figures (Control, AP, HC) to ensure visual comparability

Dietary treatments significantly altered key bacterial genera (Fig. 4). *Segatella bryantii* increased 2.1-fold under AP (log_2_FC = 1.08, *P-*adj = 0.003) and 1.9-fold under HC (log_2_FC = 0.93, *P-*adj = 0.009). *Prevotella* spp. (pooled group of multiple unresolved species within the genus *Prevotella*) increased 1.7-fold (AP, *P-*adj = 0.008) and 1.8-fold (HC, *P-*adj = 0.007), indicating increased relative abundance of *Prevotella*-associated, unresolved species groups rather than assignment to a single defined species. Conversely, *Xanthomonas* declined > threefold under both treatments (AP: log_2_FC = −1.62, *P-*adj < 0.001; HC: log_2_FC = −1.58, *P-*adj < 0.001).

Fiber-degrading genera showed treatment-specific enrichments: *Ruminococcus* increased 1.6-fold under AP (*P-*adj = 0.017), while *Fibrobacte*r increased 1.4-fold under HC (*P-*adj = 0.031). Low-abundance taxa, including *Alistipes* and *Succiniclasticum*, showed marginal reductions (*P-*adj < 0.1). Here and throughout, ‘spp.’ indicates a pooled group of multiple unresolved species within a genus, consistent with the naming conventions defined in the Bioinformatics analysis.

### Integration

Overall, taxonomic profiling revealed that agro-industrial by-products induced coordinated shifts from the phylum to the genus level. AP primarily enriched propionate-producing *Prevotella* and *Segatella* species, consistent with enhanced propanoate metabolism pathways (1.52-fold, *P* = 0.019). HC promoted diverse fiber-degrading taxa, aligning with xylanase enrichment (GH10: 1.58-fold; GH11: 1.64-fold). The phylum-level shift toward higher Bacteroidota and lower Bacillota (formerly Firmicutes), together with stable archaeal abundance, suggests diet-associated restructuring without evidence of major disruption; However, phylum-level ratios/correlations based on relative abundance are compositional and should be interpreted cautiously; in this study, mechanistic interpretation is better supported by changes in specific taxa and functional pathways linked to fermentation and hydrogen sinks.

Having established treatment-induced taxonomic shifts, the next steps were to examine whether these compositional changes translated into altered functional capacity of the rumen microbiome.

### Functional metagenomic capacity

#### Overall functional redundancy

**Fig. 5:**
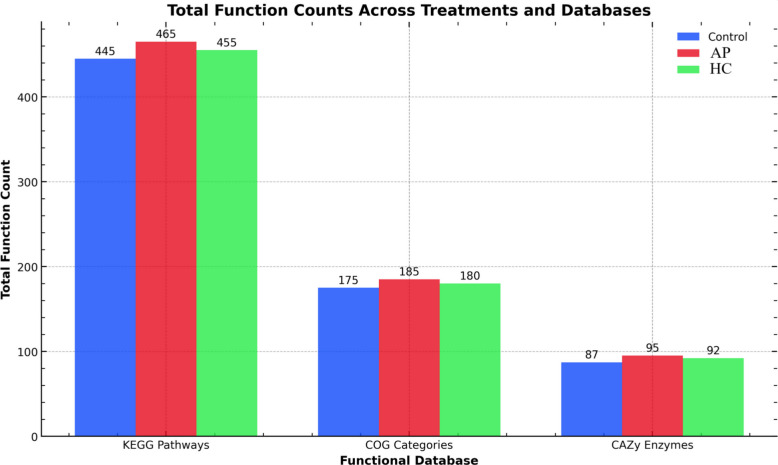
Bar graph comparing the total number of identified functional features across three annotation databases: KEGG (Kyoto Encyclopedia of Genes and Genomes) pathways, COG (Clusters of Orthologous Groups) categories, and CAZy (Carbohydrate-Active enZymes) enzyme families. Data are shown for Control (blue), Apple Pomace (AP) red color, Hempseed Cake (HC) green color. Numbers above bars indicate absolute counts of detected functions. KEGG pathways showed the highest functional diversity (445–465 pathways), followed by COG categories (175–185 categories) and CAZy enzymes (87–95 enzyme families), with minimal variation among dietary treatments indicating stable functional capacity of the rumen microbiome despite taxonomic shifts. Treatment colors are consistent across all figures (Control, AP, HC) to ensure visual comparability

A total of 445–465 KEGG pathways, 175–185 COG categories, and 87–95 CAZy enzyme families were identified across treatments. Despite taxonomic shifts, the overall functional capacity of the rumen microbiome remained stable, demonstrating high metabolic redundancy. This indicates that the microbial community maintained its ability to perform essential metabolic functions even as its composition changed (Fig. 5).

### Enrichment of carbohydrate-active enzymes and functional categories

Comprehensive functional profiling of the rumen microbiome revealed distinct effects of AP and HC supplementation on both CAZy families and COG functions.

### CAZy family enrichment

**Fig. 6:**
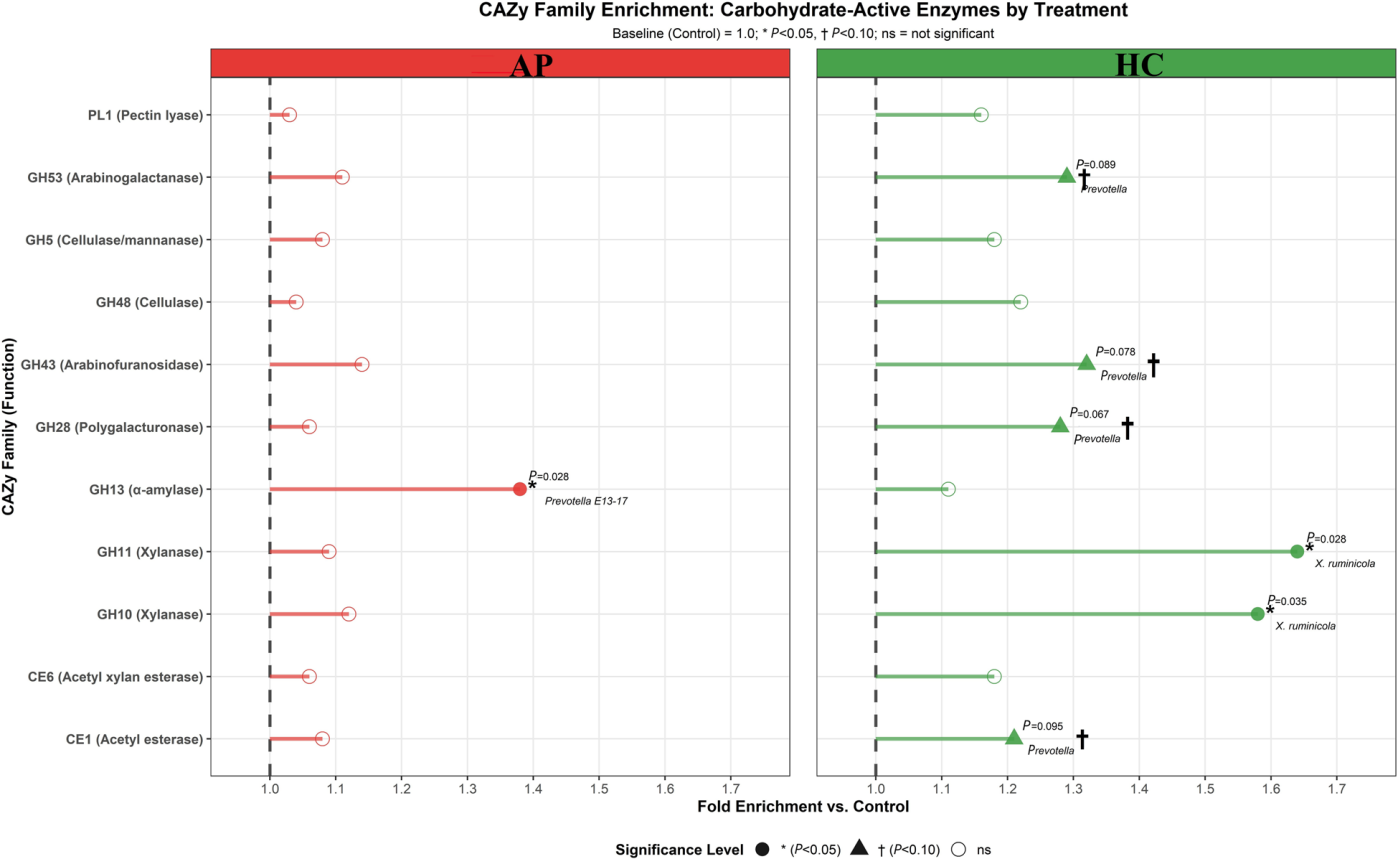
Snake plot showing carbohydrate-active enzyme (CAZy) family enrichment under AP and HC treatments versus Control baseline (1.0 ×). Separated panels display treatment-specific responses. Significant enrichments (*P* < 0.05): HC upregulated xylanases GH10 (1.58 ×, *Xylanibacter ruminicola*) and GH11 (1.64 ×); AP enriched α-amylase GH13 (1.38 ×, *Prevotella* sp.* E13-17*). Apple Pomace (AP) red color, Hempseed Cake (HC) green color. The *y*-axis shows fold change vs. Control. Microbial labels in parentheses are reported at the lowest resolved rank available (genus-level “sp.” where species is unresolved; species-level names where identified). Treatment colors are consistent across all figures (Control, AP, HC) to ensure visual comparability

AP supplementation significantly enriched GH13 (α-amylase) (1.38-fold, *P* = 0.028), consistent with increased starch-processing potential, with contributions primarily attributed to* Prevotella* sp. E13-17. In contrast, HC supplementation led to a pronounced upregulation of fiber-degrading enzymes: GH10 (xylanase) (1.58-fold, *P* = 0.035) and GH11 (xylanase) (1.64-fold, *P* = 0.028), both of which were driven by *Xylanibacter ruminicola*, reflecting improved breakdown of hemicellulose and xylan. HC also induced marginal enrichment in GH28 (polygalacturonase, 1.28-fold, *P* = 0.067) and GH43 (arabinofuranosidase, 1.32-fold, *P* = 0.078), both of which are associated with *Prevotella* spp., suggesting the emergence of pectin and arabinan utilization. Additional trends for HC included GH48 (cellulase, 1.22-fold, *P* = 0.112) and GH53 (arabinogalactanase, 1.29-fold, *P* = 0.089), indicating broader polysaccharide degradation potential. AP treatment showed no significant or marginal effects on other CAZy families (Fig. 6) (Additional file 6).

### COG functional analysis

**Fig. 7:**
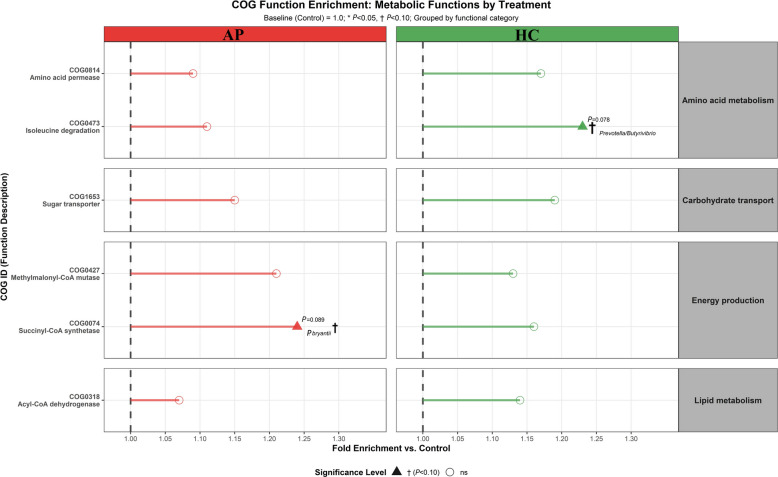
Snake plot illustrating Clusters of Orthologous Groups (COG) functional enrichment across metabolic categories under dietary treatments. Panels separate AP and HC effects by functional category. Marginally significant (*P* < 0.10): AP enriched COG0074 succinyl-CoA synthetase (1.24 ×, *Prevotella bryantii*); HC enriched COG0473 isoleucine degradation (1.23 ×, *Prevotella/Butyrivibrio*). Most functions showed modest, non-significant enrichments. Apple Pomace (AP) red color, Hempseed Cake (HC) green color. The *y*-axis shows fold change vs. Control. Microbial labels are reported at the lowest resolved rank available (species where resolved; otherwise, genus-level). Treatment colors are consistent across all figures (Control, AP, HC) to ensure visual comparability

COG profiling revealed that HC supplementation marginally increased isoleucine degradation (COG0473, 1.23-fold, *P* = 0.07), mediated by *Prevotella* and *Butyrivibrio* species, suggesting a shift in amino acid metabolism. Other COG functions, including succinyl-CoA synthetase (COG0074), methylmalonyl-CoA mutase (COG0427), sugar transporters (COG1653), and amino acid permeases (COG0814), showed only modest, non-significant changes (all *P* > 0.10), indicating stable central metabolic fluxes (Fig. 7) (Additional file 7).

### KEGG pathway analysis

**Fig. 8:**
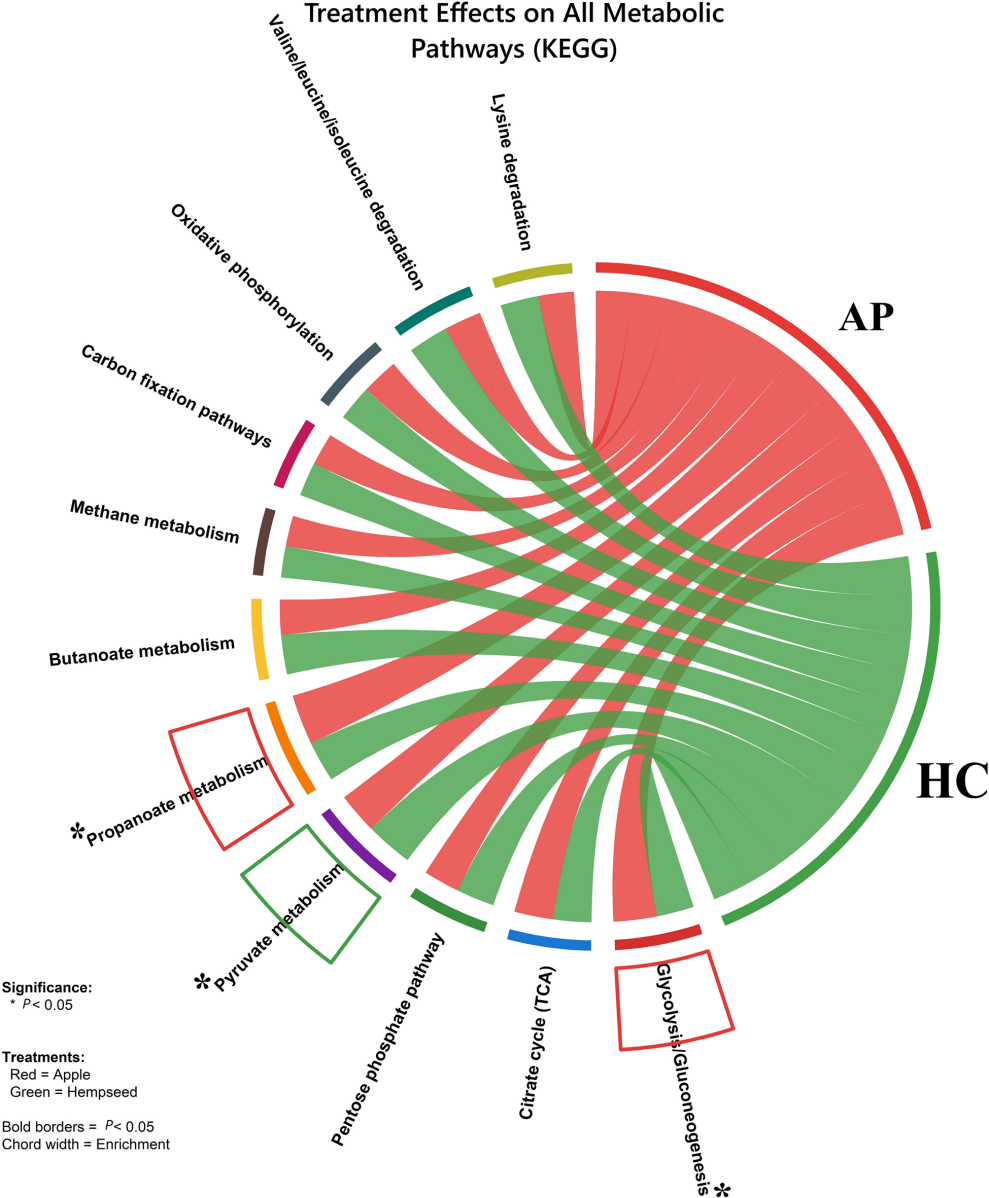
Chord diagram showing KEGG metabolic pathway enrichment in response to AP (red) and HC (green) dietary treatments versus control baseline. Chord thickness represents fold-enrichment levels. Significant enrichments (*P* < 0.05, bold colored borders); AP enriched propanoate metabolism (1.52-fold, *P* = 0.019) and glycolysis/gluconeogenesis (1.31-fold, *P* = 0.042); HC enriched pyruvate metabolism (1.42-fold, *P* = 0.041). Apple Pomace (AP) red color, Hempseed Cake (HC) green color. Treatment colors are consistent across all figures (Control, AP, HC) to ensure visual comparability

**Fig. 9:**
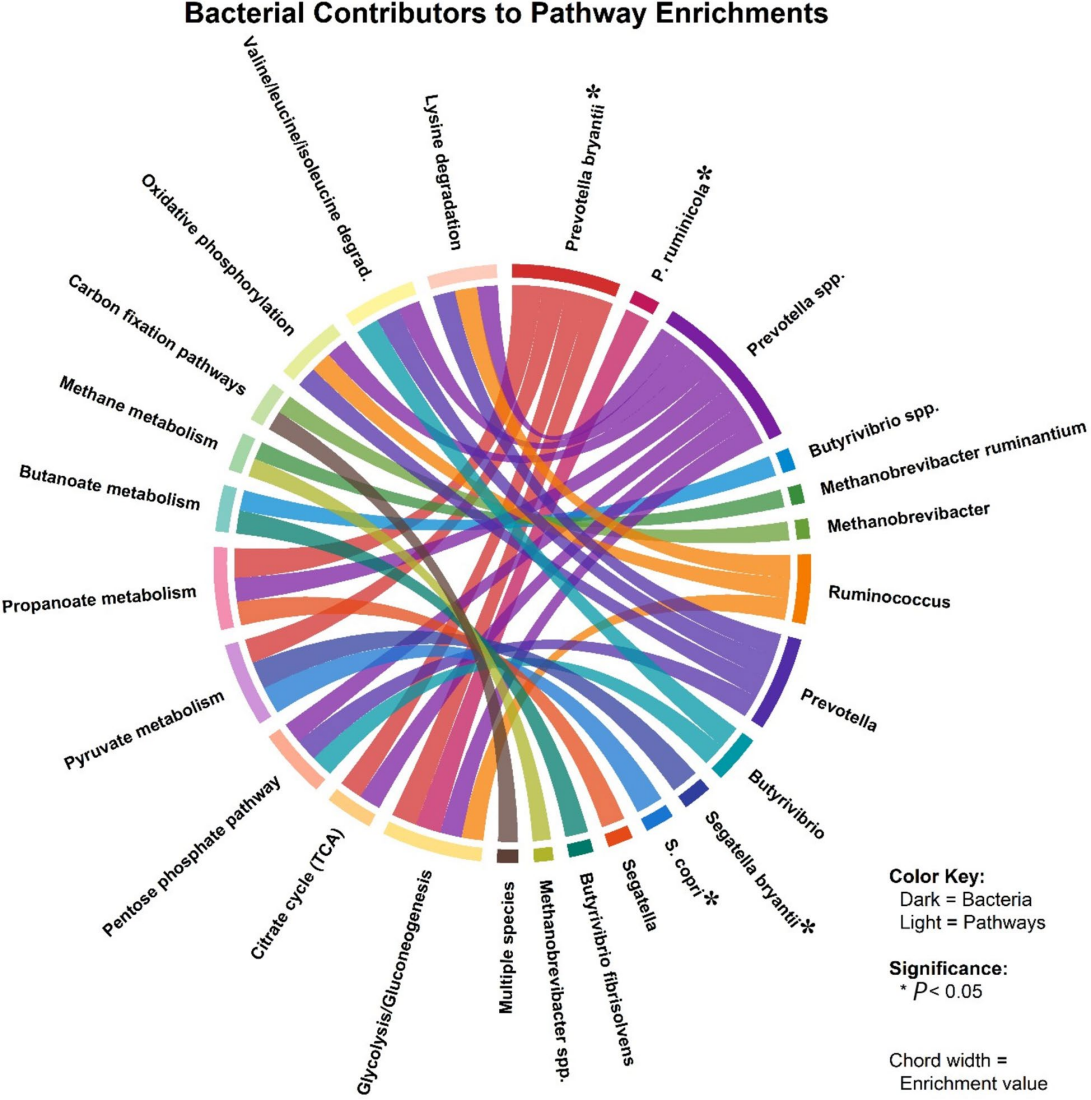
Chord diagram illustrating bacterial genera (Right side) responsible for enriching KEGG metabolic pathways (Left side) under dietary treatments. Chord width represents pathway enrichment values. Significant bacterial-pathway associations (*P* < 0.05): *Prevotella bryantii* drove propanoate metabolism and glycolysis enrichment under AP treatment; *Segatella bryantii* and *Segatella copri* enriched pyruvate metabolism under HC treatment. Apple Pomace (AP) red color, Hempseed Cake (HC) green color. The right side of the diagram represents genus-level taxa. Treatment colors are consistent across all figures (Control, AP, HC) to ensure visual comparability

Shotgun metagenomic analysis of ruminal microbiomes demonstrated that dietary supplementation with AP and HC induced distinct shifts in core metabolic pathways. AP treatment significantly enriched propanoate metabolism (1.52-fold, *P* = 0.019) and glycolysis/gluconeogenesis (1.31-fold, *P* = 0.042), primarily mediated by *Prevotella bryantii* and *Prevotella ruminicola*. These changes coincided with lower methane intensity and a lower acetate-to-propionate ratio under AP, consistent with altered fermentation patterns and potential redirection of reducing equivalents away from methanogenesis. In contrast, HC supplementation significantly upregulated pyruvate metabolism (1.42-fold, *P* = 0.041), with *Segatella bryantii* and *Segatella copri* as key contributors, consistent with altered central carbohydrate fermentation. Marginally significant effects (0.05 ≤ *P* < 0.10) were observed for AP on pyruvate metabolism (1.24-fold, *P* = 0.067) and the TCA cycle (1.18-fold, *P* = 0.089), both involving *Segatella bryantii*, and for HC on valine/leucine/isoleucine degradation (1.21-fold, *P* = 0.089) with *Prevotella* and *Butyrivibrio* contributions. Other pathways, including butanoate metabolism, methane metabolism, carbon fixation, oxidative phosphorylation, pentose phosphate pathway, and lysine degradation, showed no significant enrichment (*P* ≥ 0.10) under either treatment (Figs. 8 and 9). A complete list of KEGG pathway abundances and differential enrichment statistics (including fold changes and FDR-adjusted *P*-values) is provided in Additional file 8.

### Microbiome–phenotype relationships (descriptive associations)

**Fig. 10:**
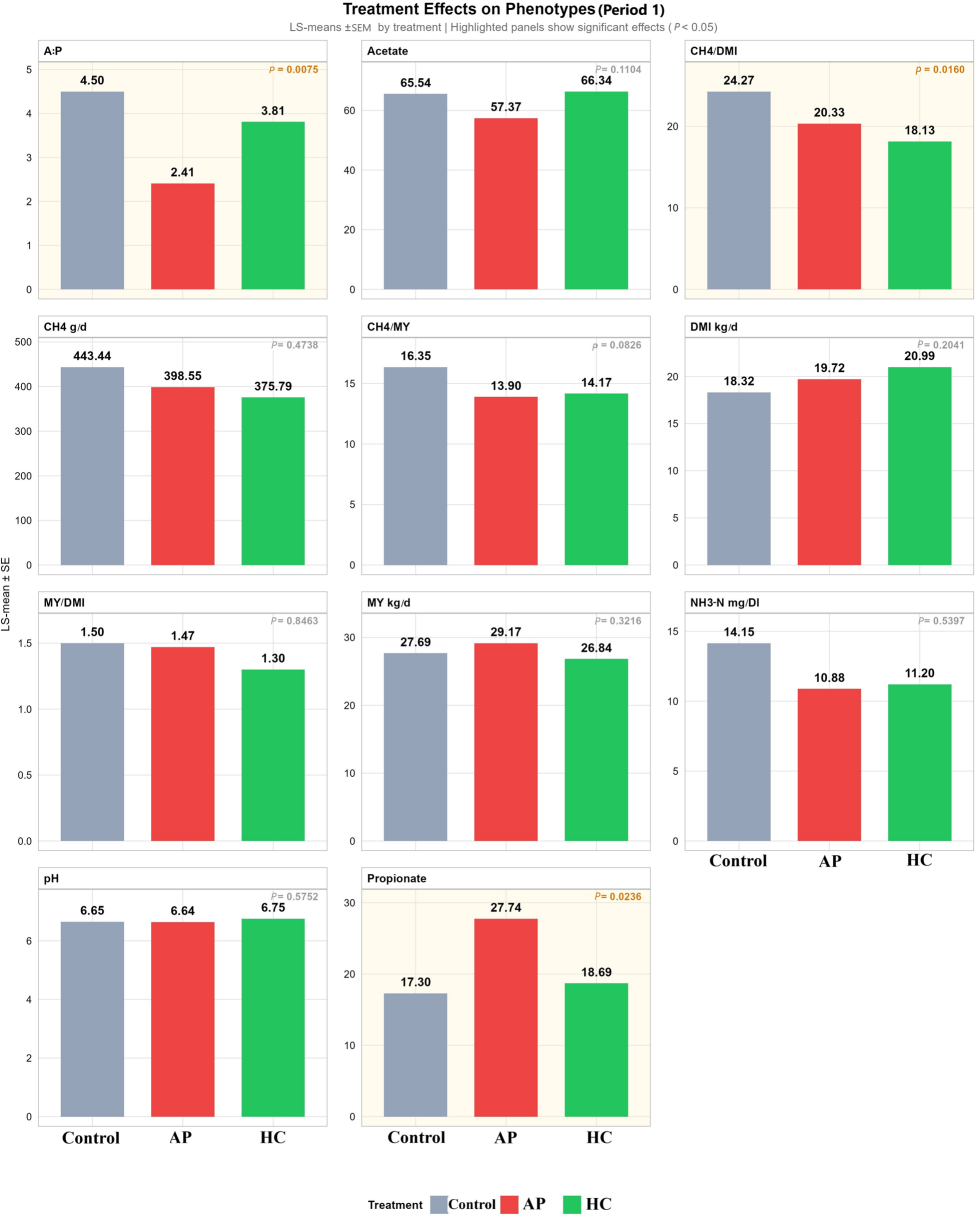
Effects of treatment on fermentation parameters and production phenotypes. Heatmap showing significant differences in the acetate-to-propionate ratio, propionate concentration, CH₄ emissions, and other fermentation parameters among the Control, AP, and HC treatments. Methane intensity (CH_4_/DMI) is expressed as g/kg DMI. statistical significance indicated by symbols (^*^P < 0.05, ^**^P < 0.01, ^***^P < 0.001). DMI = Dry matter intake; MY = Milk yield, A/P = Acetate/propionate. Apple pomace (AP) red color, hempseed cake (HC) green color. CH_4_ intensity is expressed as g/kg DMI; DMI and MY are kg/d; VFA concentrations are reported as mmol/L; ratios are unitless. Values displayed are treatment LS-means (estimated marginal means) ± SEM; significance is based on the model contrasts described in Statistical Analysis. Treatment colors are consistent across all figures (Control, AP, HC) to ensure visual comparability. All phenotypic values in this figure are derived from period 1 of the experimental design

Because this study is based on a single end-point sampling time and relative-abundance metagenomic profiles, the relationships described here are presented as descriptive associations rather than causal effects. In parallel with the observed diet-associated taxonomic and functional shifts (e.g., enrichment of *Segatella*/*Prevotella* taxa and propanoate-/carbohydrate-related functional capacity), the AP treatment reduced the acetate-to-propionate (A/P) ratio (*P* = 0.0075) and methane intensity (CH_4_/DMI; *P* = 0.016), while no significant differences were detected among treatments for total methane output, dry matter intake, milk yield, rumen pH, acetate concentration, or ammonia-N (*P* > 0.05). Values shown in Fig. 10 represent treatment LS-means (model-adjusted means), and *P*-values are derived from the corresponding treatment-effect tests/contrasts as described in the Statistical Analysis. The phenotypic least‑squares means presented here (Fig. 10) are from Period 1 of the study.

## Discussion

This study employed shotgun metagenomic sequencing to investigate the effects of dietary inclusion of AP and HC on the rumen microbiome, fermentation, methane production, and performance. Both agro-industrial by-products induced clear taxonomic and functional shifts, characterized by enrichment of propionate-producing and fiber-degrading bacteria. AP mainly enhanced starch-degrading capacity via *Prevotella*-related taxa, whereas HC increased fiber degradation through xylanase-mediated activity by *Xylanibacter ruminicola*. These microbial changes were associated with higher propionate production, increased abundance of carbohydrate-active enzymes, improved nutrient digestibility, and reduced methane emissions, without compromising milk yield (Fig. 10).

The most prominent taxonomic response was the enrichment of *Segatella bryantii*, which increased 2.1-fold with AP and 1.9-fold with HC. This species plays a key role in pyruvate metabolism, linking glycolysis to pathways involved in propionate formation, and is therefore consistent with the observed fermentation shifts and methane-mitigation phenotype. Beyond *Segatella bryantii*, pooled *Prevotella* spp. increased 1.7- and 1.8-fold with AP and HC, respectively, consistent with recent reports that *Prevotella* is central to propionate synthesis and feed efficiency in dairy cows [35]. These findings align with studies showing that wheat inclusion lowered methane by enriching propionate-producing taxa such as *Prevotella* [36], and that low-methane, efficient microbiomes are enriched in propionate- and succinate-producing bacteria [37], but our work identifies *Segatella bryantii* as a key responding species at high taxonomic resolution.

### Fiber-degrading specialists and functional guilds

By-product composition drove distinct responses among fiber-degrading specialists. Under AP, Ruminococcus spp. increased 1.6-fold, consistent with enhanced cellulose degradation; Ruminococcus is a major cellulolytic genus that forms cellulosomes to attack crystalline cellulose and likely adapts to the pectin-cellulose matrix of AP [11]. In contrast, HC supplementation preferentially enriched *Fibrobacter* spp. (1.4-fold), a key cellulolytic genus associated with high-fiber diets and plant cell wall polysaccharide degradation [8]. The strongest response was seen for *Xylanibacter ruminicola*, a hemicellulose specialist, which was markedly enriched under HC and drove 1.58-fold and 1.64-fold increases in GH10 and GH11 xylanases, respectively [10]. Together, these patterns indicate that AP and HC may select for distinct cellulolytic strategies and corresponding enzyme families, reflecting differences in fiber structure and accessibility. They also support recent Metagenome-Assembled Genome-based evidence that efficient, low-methane microbiomes are enriched in fiber degraders with metabolic traits favoring alternative hydrogen utilization [37]. Other taxa also showed metabolically relevant shifts. *Segatella copri*, a close relative of *Segatella bryantii*, contributed to pyruvate metabolism enrichment under HC, suggesting functional redundancy within the genus [38]. *Butyrivibrio* spp., involved in fiber degradation and biohydrogenation of unsaturated fatty acids, were linked to enrichment of isoleucine degradation pathways, particularly relevant given the high unsaturated fatty acid content of HC [39, 40]. Conversely, *Xanthomonas* spp. likely transient, non-core rumen bacteria, declined by more than threefold under both treatments, indicating that altered substrate and fermentation conditions were unfavorable for this genus. Minor decreases in *Alistipes* and *Succiniclasticum* (*P* < 0.1) [35, 41] were observed, though their biological relevance remains uncertain.

### Phylum-level shifts and the Bacillota/Bacteroidota ratio

Both AP and HC increased Bacteroidota and decreased Bacillota, lowering the Bacillota/Bacteroidota ratio and indicating substantial restructuring of the rumen community. Similar reductions in Bacillota/Bacteroidota ratios have been reported when barley and soybean meal are replaced with by-product, based concentrates in RUSITEC systems, accompanied by increases in Proteobacteria and Succinivibrionaceae [42]. While some studies link lower Bacillota/Bacteroidota ratios to improved feed efficiency, comparative metagenomics suggests that functional potential and specific taxa, rather than the ratio itself, drive host performance [15]. Recent microbiome, metabolome GWAS work further shows that Holstein lactation performance is associated with heritable carbohydrate metabolism across both Bacteroidota and Bacillota taxa [12]. In this context, the observation that Bacillota/Bacteroidota ratios decreased while production was maintained or enhanced suggests that the enrichment of propionate-producing Bacteroidota (e.g., *Segatella bryantii* and *Prevotella* spp.) is more consequential than changes in the overall phylum-level ratio itself.

Notably, because phylum abundances are presented as relative proportions, apparent negative correlations between dominant phyla can arise from compositional constraints and should not be interpreted as evidence of direct biological antagonism. Importantly, evidence across studies is not uniform regarding whether a lower the Bacillota/Bacteroidota ratio (formerly F/B) consistently corresponds to lower methane emissions or improved efficiency, and the ratio itself is a coarse compositional summary rather than a functional mechanism. Accordingly, we interpret the reduced Bacillota/Bacteroidota ratio as a result of community restructuring under AP/HC rather than as a direct cause of methane mitigation. In this dataset, the biologically stronger signals relate to specific taxa and functions, particularly enrichment of *Segatella*/*Prevotella* taxa and propanoate-/pyruvate-related functional capacity (KEGG), as well as CAZy shifts linked to carbohydrate utilization, consistent with altered fermentation patterns (e.g., higher propionate and lower A/P ratio under AP).

### Methanogens and alternative hydrogen sinks

Methanogenic archaeal relative abundances remained largely unchanged across treatments, while methane intensity decreased; this pattern is consistent with mitigation via altered fermentation and hydrogen allocation rather than direct suppression of methanogens, but it does not provide direct physiological evidence of hydrogen redirection. This contrasts with studies where unsaturated fatty acids reduced Methanobrevibacter abundance [43] or where 3-nitrooxypropanol caused large declines in methanogens and methanogenesis gene expression [16], and with interventions such as *Bacillus subtilis* plus *Macleaya cordata* extract that lowered specific Methanobrevibacter spp. alongside methane [44]. The findings of our study may more closely resemble mechanisms where methane inhibition is achieved by redirecting hydrogen flux, as seen when different mitigation strategies (e.g. oils vs. inhibitors) differentially affect methanogen abundance versus hydrogen-cycling networks [45].

Functional metagenomic results support a hydrogen redirection mechanism. AP particularly enriched propanoate metabolism (1.52-fold) via the succinate-propionate pathway, largely mediated by *Segatella bryantii*, *Prevotella ruminicola* and related taxa. This pathway (fumarate—succinate—propionate) serves as a major alternative hydrogen sink, directly competing with methanogenesis for reducing equivalents. However, ruminal H₂ concentration was not measured in this study, and hydrogen-cycling activity was not directly quantified; therefore, increased hydrogen capture via propionate formation is inferred from the fermentation profile and metagenomic functional enrichment rather than verified by direct physiological measurement. Genes encoding key enzymes (e.g. fumarate reductase, methylmalonyl-CoA mutase) were overrepresented, along with glycolysis/gluconeogenesis (1.31-fold) and pyruvate metabolism (1.42-fold) under AP and HC, respectively, indicating enhanced central carbohydrate metabolism to sustain higher VFA production and hydrogen use. These results parallel mechanistic work showing wheat supplementation reduces methane via increased propionate production and hydrogen consumption [36] and that low-methane microbiomes are enriched in succinate producers and efficient fiber degraders [37]. Our study extends these observations by identifying specific species (*Segatella bryantii*, *Prevotella ruminicola*, *Prevotella* sp. E13-17) and pathways (propanoate, pyruvate, glycolysis) underpinning increased alternative hydrogen sinks with AP and HC. Fiber degraders likely contribute to hydrogen remodeling as well. Enrichment of *X. ruminicola*, *Ruminococcus* and *Fibrobacter* not only enhanced fiber breakdown but may have shifted hydrogen flow within the community. Seaweed-based methanogenesis suppression has been associated with increased transcription of hydrogenases in hydrogen-consuming bacteria and reduced transcription of methanogenesis genes [46], suggesting that community-wide hydrogen cycling, rather than methanogen loss alone, is critical. It is possible that AP and HC may promote a community configuration in which propionate producers and fiber-degrading taxa collectively increase hydrogen utilization via non-methanogenic pathways. However, our archaeal assessment is based on metagenomic relative abundance and we did not quantify methanogenesis marker genes (e.g., *mcrA*/heterodisulfide reductase (*hdr*)) by qPCR or metagenomic gene-centric analysis, nor did we measure pathway flux; therefore, the ‘hydrogen competition/redirection’ explanation should be interpreted as an inference rather than direct functional validation. Therefore, the hydrogen competition/redirection explanation should be interpreted as an inference based on the combined pattern of reduced CH_4_ intensity, stable methanogen relative abundance, and enrichment of propionate-associated fermentation pathways, rather than as direct validation of altered methanogenic activity. Future work should validate this mechanism by quantifying methanogenesis markers (e.g., *mcrA/hdr* by qPCR and/or metatranscriptomics) and, where feasible, directly assessing hydrogen allocation using metabolic flux approaches (e.g., ^13^C-labeled substrate tracing and/or rumen H_2_ measurements). The methane-intensity reductions observed with AP and HC should be interpreted differently from the responses reported for dedicated methane inhibitors. The magnitude of methane mitigation varies substantially among feed interventions and depends on additive type, dose, and study duration. In contrast to specialized inhibitors, AP and HC were evaluated here as agro-industrial by-product feed ingredients that can partially replace conventional dietary components while also contributing to methane-intensity mitigation. Thus, their practical value lies not only in methane reduction, but also in circular-economy use of by-products without detectable reductions in dry matter intake or milk yield in this short-term study.

### Production performance and future directions

AP and HC supplementation reduced methane emissions without compromising milk yield, indicating that environmental and economic goals can be jointly achieved through appropriate dietary strategies. This aligns with evidence that well-designed feed interventions, such as 3-nitrooxypropanol in beef cattle [16] or *Bacillus subtilis* plus *Macleaya cordata* extract in dairy cows [44], can lower methane with minimal or positive effects on performance. In our study, enrichment of *Segatella bryantii*, *Prevotella* spp., and propanoate metabolism likely supported gluconeogenesis and lactose synthesis via increased propionate supply, while the protein (30%−40% crude protein) and fatty acids in HC may have contributed to maintaining milk protein and fat. Although this work clarifies key microbial and functional shifts induced by AP and HC, several questions remain. Multi-omics approaches, including metatranscriptomics and metabolomics, will help pinpoint the active pathways and regulatory mechanisms involved in methane mitigation. In addition, future work will incorporate genome-resolved analyses using the reconstructed MAGs to support species/strain-level interpretation of diet-responsive functions and to complement the read- and gene-centric findings reported here. Identifying robust microbial and functional biomarkers could further support precision nutrition strategies aimed at simultaneously improving feed efficiency and reducing methane emissions. The integration between microbiome features and host phenotypes in this study is association-based and does not include formal quantitative microbiome–phenotype modeling (e.g., Spearman correlation screening with multiple-testing control, random forest feature selection, or constrained ordination such as RDA/CCA). Therefore, while the observed fermentation shifts and methane-intensity reductions are consistent with enrichment of propionate- and carbohydrate-utilization pathways/taxa, the present data do not allow ranking of ‘core driver’ microbes/pathways or causal attribution. Future studies should combine longitudinal sampling with absolute-abundance or activity-based measurements (e.g., qPCR/metatranscriptomics and hydrogen/methanogenesis markers) and apply multivariable modeling to prioritize robust microbial and functional predictors of methane intensity. Given the cross-sectional design and compositional nature of relative-abundance data, these approaches, ideally combined with longitudinal sampling and activity-based measurements, would be valuable in future work to prioritize candidate taxa and pathways (e.g., *Segatella bryantii*, *Xylanibacter ruminicola*, propanoate- and carbohydrate-active enzyme pathways) for mechanistic validation of methane-mitigation mechanisms.

A key design consideration is that rumen metagenomic profiling was based on a single, end-point sampling on d24. While the 17-d adaptation preceding sampling was intended to allow microbial adjustment to each diet, the lack of repeated rumen sampling prevents assessment of within-cow temporal stability and makes it difficult to distinguish persistent diet effects from short-term or transient fluctuations. Therefore, the microbiome findings should be interpreted as an end-point, cross-sectional snapshot and the microbiome–phenotype relationships as associations rather than evidence of temporal causality. Future work should use longer-duration designs with time-series rumen sampling (e.g., end of adaptation, mid-period, and end of period) to quantify the stability and trajectory of taxonomic and functional changes induced by AP and HC. Although AP and HC showed potential as agro-industrial by-product feed ingredients with methane-mitigation effects, but their commercial application depend on local availability, processing and transport costs, preservation method, nutrient variability, and price relative to conventional feed ingredients. In the present study, the inclusion levels of AP (14.3% AP/straw silage) and HC (10% HC) were selected to evaluate biological responses under controlled conditions and were not intended to define economically optimal inclusion rates. Future work should therefore assess the cost-effectiveness of AP and HC under commercial dairy-farm conditions using local feed prices, supply-chain constraints, and ration-formulation requirements.

### Limitation

Although the 24 d period (17 d adaptation/measurement plus 7 d collection) is consistent with published Latin-square feeding trials, it represents a short-term intervention and may not fully capture longer-term microbiome stabilization or adaptive rebound. Therefore, the microbial and methane responses reported here should be interpreted as short-term effects, and longer-duration studies with repeated sampling are needed to confirm their persistence. In addition, rumen metagenomic sequencing was performed on a single end-point sample (d 24), so temporal dynamics and within-cow stability of microbiome responses could not be evaluated. Additionally, we did not perform formal microbiome–phenotype correlation or multivariate modeling analyses in this manuscript; thus, microbiome features discussed in relation to methane intensity and fermentation should be interpreted as qualitative associations rather than statistically ranked drivers.

A methodological limitation is that while host DNA was removed by mapping reads to the bovine genome, we did not perform explicit subtraction against multiple feed/plant genomes due to the mixed-ingredient nature of the diets and the risk of reference-driven bias. Consequently, the proportion of residual plant-derived DNA within the non-host reads was not quantified; if present, it would primarily reduce effective microbial sequencing depth and should be considered when interpreting microbiome results, particularly for low-abundance taxa and pathway-level inferences. Finally, although MAGs were reconstructed and quality-assessed, we did not conduct downstream genome-resolved comparative analyses; therefore, MAG-based species/strain-level functional gene inventories are not presented in this manuscript. In addition, we did not quantify methanogenesis marker genes (e.g., *mcrA*) by qPCR or via a dedicated metagenomic gene-centric analysis; therefore, functional changes in methanogens were not directly assessed. As DMI and milk yield were summarized over the 7-d collection phase, the study may have limited power to detect small-to-moderate treatment effects on production traits. Thus, the non-significant differences in DMI and MY should be interpreted cautiously and validated in longer-duration trials.

## Conclusions

**Fig. 11:**
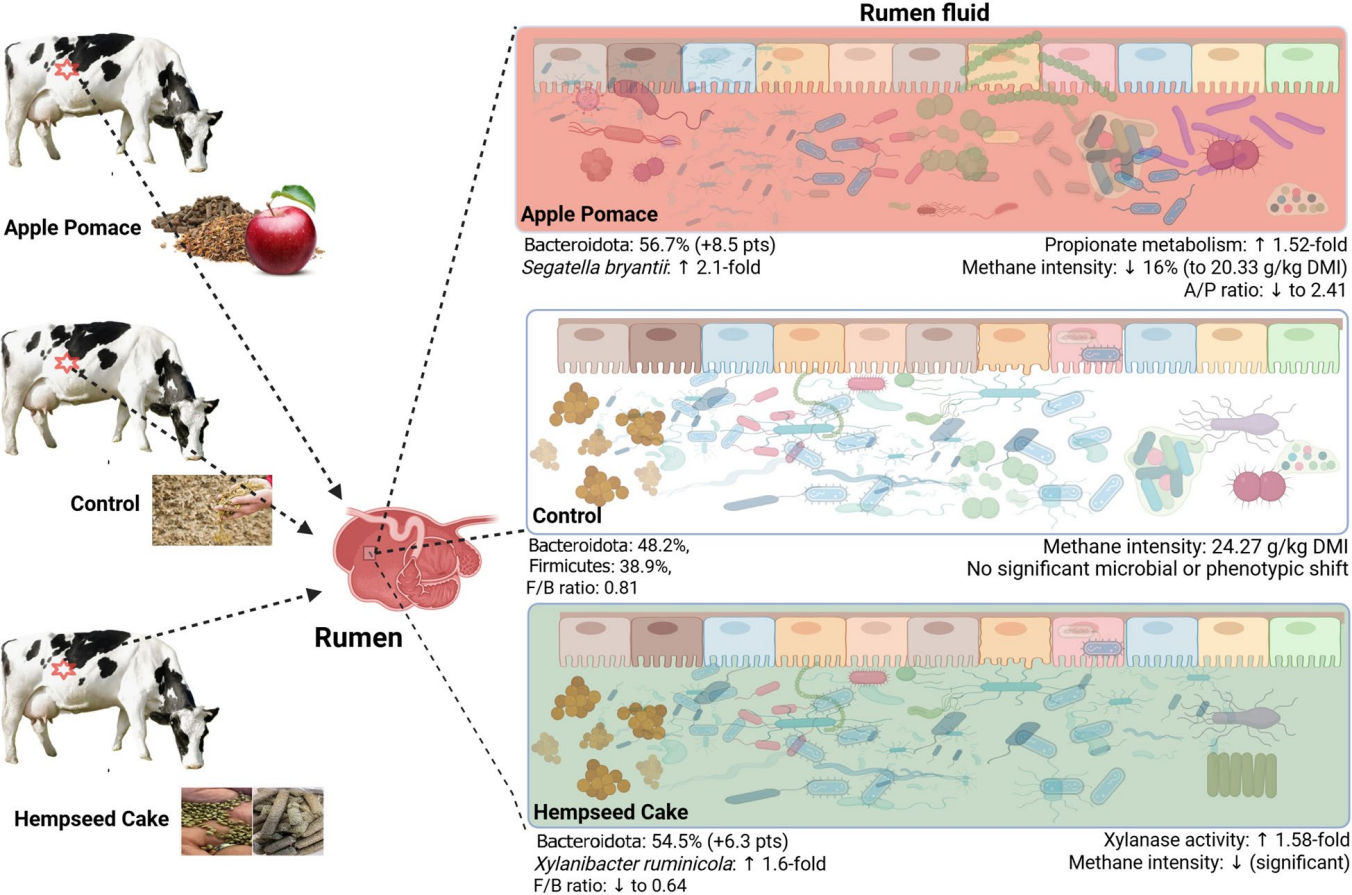
Integrated graphical summary of diet-dependent changes in rumen microbial composition, functional pathways, fermentation characteristics, and methane intensity in cows fed control, apple pomace (AP), or hempseed cake (HC) diets

In this short-term (24-d) controlled study in mid-lactation Holstein cows, incorporating AP (14.3% apple pomace/straw silage) or HC (10% hempseed cake) lowered methane intensity (CH_4_/DMI) while maintaining dry matter intake and overall rumen functional capacity. Within this short-term experiment, AP was associated with enrichment of *Segatella*/*Prevotella* taxa and carbohydrate/propanoate-related functional signals (including GH13), whereas HC was associated with enrichment of fiber-degrading taxa (e.g., *Xylanibacter*, *Fibrobacter*) and xylanase families (GH10, GH11). Both by-products shifted the community toward higher Bacteroidota and a lower Bacillota/Bacteroidota ratio, which we interpret as a descriptive outcome of microbial restructuring rather than a direct cause of methane mitigation. The functional and fermentation changes (e.g., propionate-associated pathways/taxa and CAZy shifts) provide a more biologically informative basis for the observed reduction in methane intensity (Fig. 11). In addition, the inclusion levels of AP and HC were determined in an in vitro trial, and a 10% inclusion level was used in this feeding experiment [47]. AP and HC are promising sustainable feed alternatives that reduced methane intensity in this short-term study while maintaining intake and production; the combined fermentation and metagenomic patterns are consistent with a shift toward alternative hydrogen sinks, but methanogenesis marker genes (e.g., *mcrA*) were not quantified and should be validated in future work. In this short-term study, AP and HC reduced methane intensity without detectable changes in dry matter intake or milk yield. Repurposing these agro-industrial by-products also minimizes waste-disposal costs, aligning with circular-economy principles [22, 47]. These findings support AP and HC as promising by-product ingredients for methane-intensity mitigation strategies in mid-lactation Holstein cows under controlled feeding conditions; validation is needed in longer-duration studies and commercial farm settings before broader generalization.

## Supplementary Information

Additional file 1: Experimental workflow for dairy cow study.

Additional file 2: Alpha diversity metrics.

Additional file 3: Taxonomic Diversity.

Additional file 4: Overall Functional Redundancy.

Additional file 5: Effect sizes (Δ, % change, and standardized mean differences) for key microbial and phenotypic outcomes.

Additional file 6: CAZy Family Enrichment.

Additional file 7: COG Functional Analysis.

Additional file 8: KEGG Pathways Analysis.

## Data Availability

The datasets are included in this article and available from the corresponding author on reasonable request.
