# Supplementary figures and images for: Apple pomace and hempseed cake can reduce methane intensity (CH₄/DMI) and alter the rumen microbiome in dairy cows: a shotgun metagenomic approach
Source: J Anim Sci Biotechnol. 2026 Jun 24;17:128. doi: 10.1186/s40104-026-01448-1 (PMC13292333; doi:10.1186/s40104-026-01448-1)

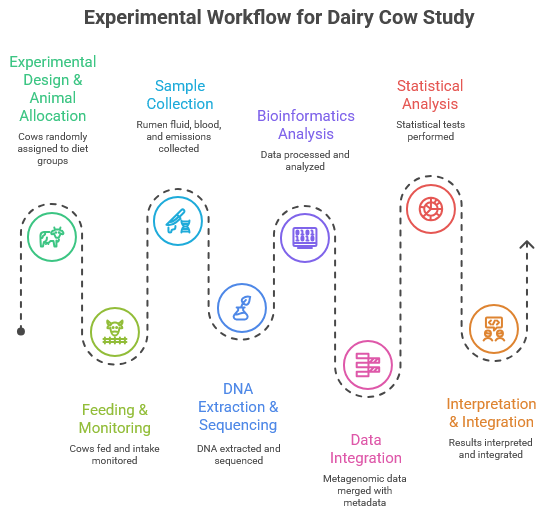

Supplement: Supplementary file 1 — Additional file 1: Experimental workflow for dairy cow study. [file 40104_2026_1448_MOESM1_ESM.png]
